# Establishment of an ultrasound-guided protocol for the assessment of hip joint osteoarthritis in rabbits–A sonoanatomic study

**DOI:** 10.1371/journal.pone.0291177

**Published:** 2023-09-14

**Authors:** Inês Tomé, Sofia Alves-Pimenta, Luís Costa, José Pereira, Roberto Sargo, Hugo Brancal, Mário Ginja, Bruno Colaço

**Affiliations:** 1 Department of Veterinary Sciences, University of Trás-os-Montes e Alto Douro, Vila Real, Portugal; 2 CECAV, Centre for Animal Sciences and Veterinary Studies, Associate Laboratory for Animal and Veterinary Science—AL4AnimalS, University of Trás-os-Montes e Alto Douro, Vila Real, Portugal; 3 Department of Animal Science, University of Trás-os-Montes e Alto Douro, Vila Real, Portugal; 4 Clínica Veterinária da Covilhã, Covilhã, Portugal; University of Life Sciences in Lublin, POLAND

## Abstract

Ultrasound (US) has emerged as one of the most applied imaging tools to diagnose musculoskeletal disorders and assist in guided intra-articular administrations. Nevertheless, in evaluating the rabbit hip joint, there is a need for an ultrasonographic approach. Therefore, this study aimed to describe the hip sonoanatomy, develop and validate a US-guided protocol to assess the hip joint in rabbits and apply this protocol *in vivo*. This study was carried out in three phases, phase I: a pilot cadaveric study, to assess the applicability of different US approaches in the hip of rabbits and, consequently, develop a detailed US-guided protocol (2 rabbit cadavers, n = 4 hips); phase II: validation of the established US-guided protocol through a numerical scoring system in healthy joints (11 rabbit cadavers, n = 22 hips), and, lastly, phase III: application of the US-guided protocol *in vivo* in osteoarthritic joints (5 rabbits, n = 5 hips). A total of six planes were validated, two in the ventral approach and four in the dorsal approach. The ventral transverse plane was deemed more informative regarding the hip joint sonoanatomy, enabling the identification of a greater number of structures when compared to the other planes. Nevertheless, this study suggested that the isolated application of a plane was deemed insufficient for a complete and detailed evaluation of the hip joint anatomy, rendering it necessary to employ other planes complementarily. Furthermore, the established US-guided protocol allowed a definitive diagnosis of OA, and osteophytes and capsular hypertrophy were among the defects most frequently detected. This novel study provided US anatomical landmarks for forthcoming therapeutic research and monitoring of OA development, granting the accurate identification of osseous and cartilaginous defects.

## Introduction

To date, the rabbit, as an animal model, has been extensively applied in osteoarthritic research [1, 2], allowing a foreseeable translation of findings to the clinical scenario [3]. Osteoarthritis (OA) represents one of the major non-inflammatory articular diseases in mammals [4] and is either described as idiopathic with no recognizable underlying cause [5, 6] or has its onset in mechanical overload [7, 8]. Joint instability can compromise the normal load of the joint surface and trigger OA [9]. Hip dysplasia is an example in which joint instability is present and is deemed a leading precursor of OA [10], being occasionally described in rabbits [11–13].

Imaging in the diagnosis and staging of OA has evolved greatly in the last few years [14, 15]. The accessibility of cost and interpretation of the radiography compared to more advanced imaging modalities like computed tomography (CT) and magnetic resonance imaging (MRI) [15], makes radiography the most used modality to track osteoarthritic progression and confirm structural defects [14, 16]. However, radiographic evaluation is restricted to the identification of joint space narrowing, osteophytes, and other subchondral changes, such as cysts and sclerosis [15, 17, 18], which mainly develop at later stages of OA [14]. In contrast, ultrasound (US) and magnetic resonance imaging (MRI) allow a sensitive and specific assessment of structural features and inflammatory involvement at an initial phase of OA [19]. Nevertheless, the flat learning curve [20, 21], the narrow acoustic window [22] and the high acoustic impedance in the bone-soft tissue interface [23] may explain the continuous resistance to US application [24], and MRI is expensive and time-demanding [14]. Also, CT admits a 3D imaging reconstruction and an excellent image resolution of the anatomical structures [14], despite the drawback of intensive exposure to ionizing radiation [15]. Overall, the US represents a cost-effective tool compared to radiography, CT and MRI, providing a real-time dynamic evaluation of bone and soft tissues with zero exposure to ionizing energy [22].

The US role is changing, and due to the latest technological enhancements, may represent one of the most promising imaging modalities [21, 25]. For instance, in infants, the US represents the gold standard in the diagnosis of hip dysplasia [26], allowing a reliable visualization of the hip in the first few months of life [27, 28]. As such, the US has the potential to be an accurate and consistent imaging tool if standardized guided approaches and grading schemes are implemented [29]. Several planes have been described in the literature regarding the hip joint assessment and US-guided injections: in the dorsal approach, the transverse planes in humans, horses and dogs [30–33], the coronal and sagittal planes in humans [33], and the caudolateral–craniomedial oblique in horses [34]; and in the ventral approach, the sagittal plane in humans [35, 36] and the transverse plane in humans and rabbits [36, 37]. Nevertheless, reliable US methodologies to assess hip OA onset and progression are currently lacking in rabbits. Therefore, the main goals of the present study were: (1) to describe the hip sonoanatomy, define landmarks for the hip US assessment, and develop a US-guided protocol to assess the hip joint; (2) to evaluate the applicability of the defined protocol in rabbit cadavers using a numerical scoring system, and (3) to validate the clinical application of the US-guided validated protocols in osteoarthritic hip joints.

## Material and methods

### Study design

This work was divided into three phases: phases I and II had a cadaveric prospective design whereas phase III had an *in vivo* prospective design. All animals were obtained from Granja San Bernardo, Navarra, Spain. The conducted procedures were followed in accordance with the European and National legislation on the protection of animals used for scientific purposes (European Directive 2010/63/EU and National Decree-Law 113/2013) and the research study was approved by the competent Portuguese authority, the Directorate-General for Food and Veterinary (DGAV_0421/000/000/2022). To minimize possible biases in this study due to uncontrolled variable effects on the results, a single researcher was assigned to perform the US assessments.

### Cadaveric study

A total of thirteen male New Zealand White rabbits with 14-week-old and free from hip abnormalities were euthanized for reasons unrelated to the present study, using pentobarbital (Eutasil^®^, CEVA Saúde Animal, Portugal, at 100 mg/Kg, IV). The cadavers were collected and frozen (within 24 hours after death) to be used in the first and second phases of the experiment. At the time of the US assessment, the cadavers were thawed at room temperature for at least 24 hours before use.

#### Phase I: Pilot stage, definition of anatomical landmarks, establishment of the ultrasound-guided protocol and hip sonoanatomy

A pilot stage was carried out in two rabbit cadavers (two rabbits, n = 4 hips) to evaluate the applicability of the potential approaches mentioned in the literature for other species in the US assessment of the rabbit hip joint. For this purpose, cadavers were positioned in dorsal or lateral recumbency to the ventral or dorsal approaches, respectively, and hair was clipped in the targeted area. US coupling gel (Mebaline, Barcelona, Spain) was applied to the skin to generate a suitable acoustic interface and appropriate scanning depth and gain were selected (1–2 centimetres and 45–55 decibels, respectively). Two foci were positioned at the level of the region of interest. Optimal probe positioning and the structures visualized in each plane were noted. The US evaluation was performed using a portable US machine (Logiq e, General Electric Medical Systems, Buc, France), equipped with a high-frequency linear transducer (L10-22-RS, General Electric Medical Systems, Buc, France), operating at 20 MHz and configured with a musculoskeletal preset. Following the initial US scanning, the sonoanatomy structures were described based on the gross anatomical dissection. The skin and subcutaneous tissue were incised from inguinal or gluteal regions respectively. The overlaying fascia was dissected, and the muscles were exposed. Moreover, when further clarification was necessary, the muscles were detached from their respective insertion site and a US scanning was performed sequentially to better acknowledge the sonoanatomy of the plane assessed.

The muscles detached whenever required were, in the ventral approach, the sartorius, pectineus, vastus medialis, rectus femoris, iliacus and psoas major, and muscles, and in the dorsal approach were the gluteofemoralis, gluteus superficialis, gluteus medius, gluteus accessorius, piriformis, and gluteus profundus muscles.

After the pilot stage, the following planes were defined. In the ventral approach, the sagittal and transverse planes were selected, whereas in the dorsal approach the dorsal, caudolateral-craniomedial oblique, caudomedial-craniolateral oblique, and transverse planes were chosen. Anatomical landmarks were also identified for each plane and a repeatable methodology for the acquisition of quality images was defined. Then, a detailed protocol for each plane and landmarks will be described for all ventral and dorsal approaches.

**Fig 1:**
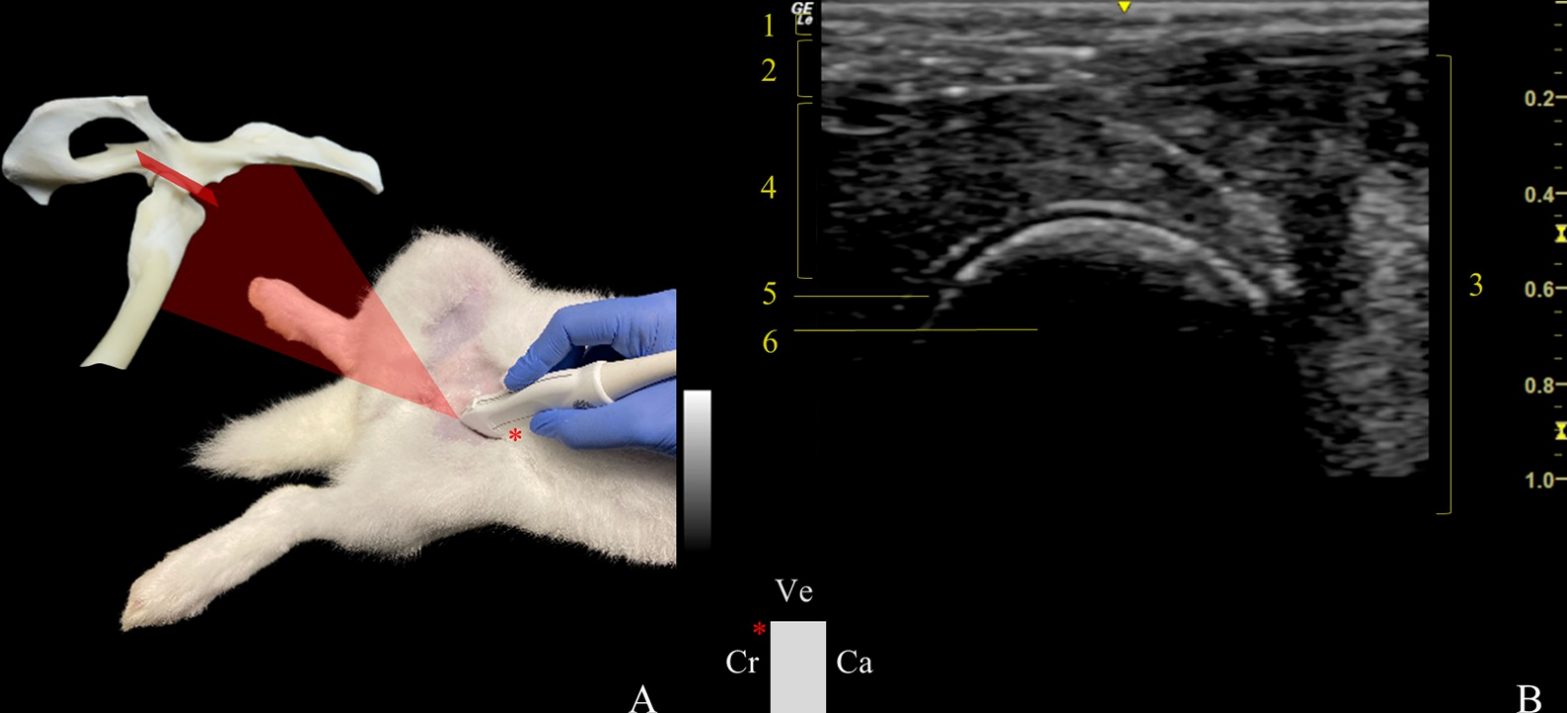
Transducer positioning and resulting ultrasound image obtained in a ventral approach in the sagittal plane of the left hip joint. A: Rabbit placement in dorsal recumbency with the probe at the level of the joint, and corresponding anatomic specimen. The red rectangle and asterisk represent the probe orientation and indicator, respectively. B: Sonoanatomy of the hip: (1) skin and subcutaneous tissue, (2) sartorius muscle, (3) vastus medialis muscle, (4) rectus femoris muscle, (5) articular cartilage of the femoral head, and (6) femoral head. Ve: ventral, Cr: cranial, and Ca: caudal.

In the **ventral approach**, the rabbit was placed in dorsal recumbency with the hindlimbs in a neutral position and the planes were assessed as follows:

*Sagittal plane*. In the ventral approach sagittal plane (VSP) (Fig 1), the probe was set parallel to the medial aspect of the femoral diaphysis and moved proximally. Once the hip joint was reached, the probe was turned 90˚, so that it laid perpendicularly to the femoral diaphysis and, then tilted laterally ~30˚, until the acetabulum and femoral head were fully centred in the visual field. When the transducer was slid from the joint to the femoral shaft, it allowed the visualization of the ventral aspect of the femur, femoral neck, and the ventral margin of the acetabulum. The femoral diaphysis was the anatomical landmark taken into consideration when accessing the hip VSP and the indicator on the probe was pointed cranially.

**Fig 2:**
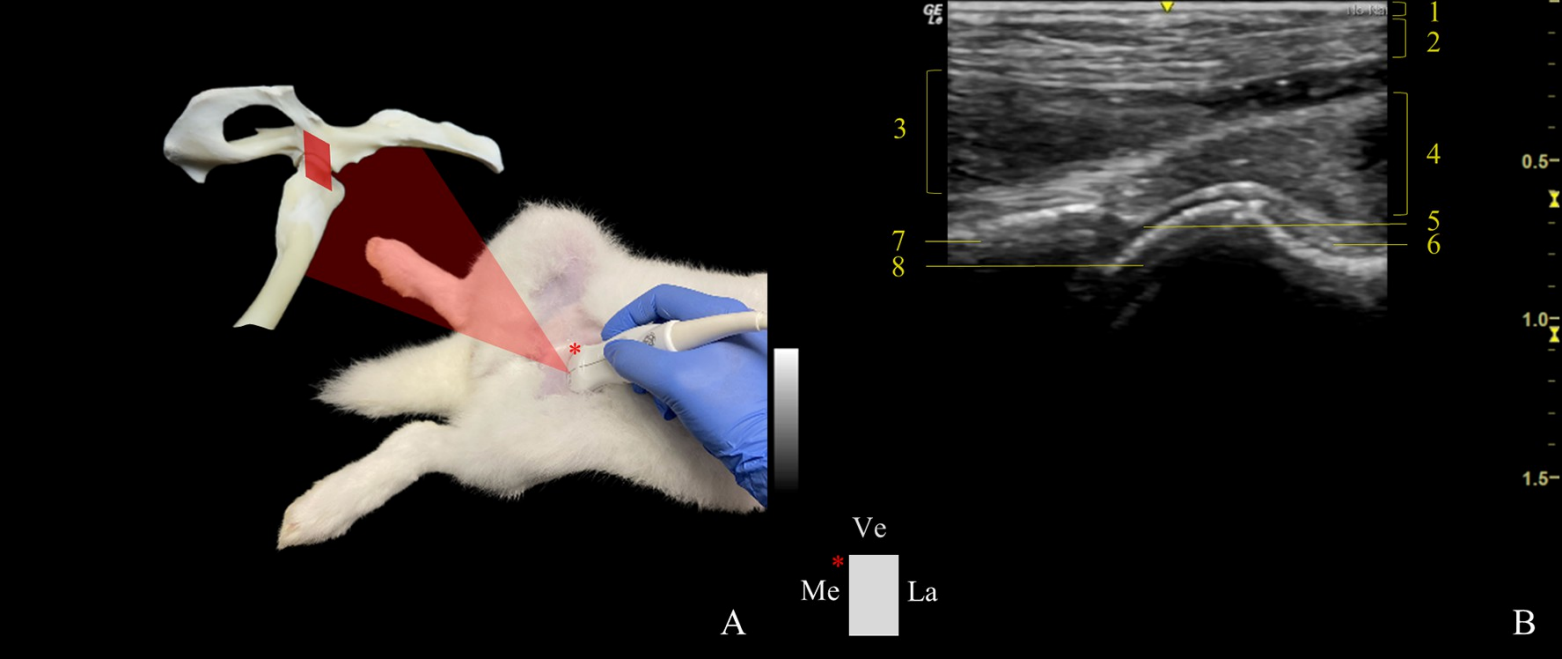
Transducer positioning and resulting ultrasound image obtained in a ventral approach in the transverse plane of the left hip joint. A: Rabbit placement in dorsal recumbency with the probe at the level of the joint, and corresponding anatomic specimen. The red rectangle and asterisk represent the probe orientation and indicator, respectively. B: Sonoanatomy of the hip: (1) skin and subcutaneous tissue, (2) sartorius muscle, (3) rectus femoris muscle, (4) iliacus and psoas major muscle, (5) articular cartilage of the femoral head, (6) ventral aspect of the articular capsule, (7) acetabulum, and (8) femoral head. Ve: ventral; Me: medial, and La: lateral.

*Transverse plane*. In the ventral approach transverse plane (VTP) (Fig 2), the probe was set parallel to the femoral diaphysis, the main landmark, and then moved proximally. The hyperechoic image of the femoral diaphysis was then followed by the femoral neck and towards the femoral head. As soon as the hip joint was reached, the transducer was tilted cranially ~30˚ until allowing the visualization of the acetabulum, femoral head, and neck. When the transducer was slid from the cranial to the caudal ventral acetabular rim, it admitted the complete assessment of the ventral acetabular margin and aspect of the femoral head, neck, and joint capsule. To evaluate the round ligament integrity, a dynamic examination was required, and distraction force was applied to the distal aspect of the femur, repeatedly, until the ligament was recognized. The probe was placed facing the acetabulum.

**Fig 3:**
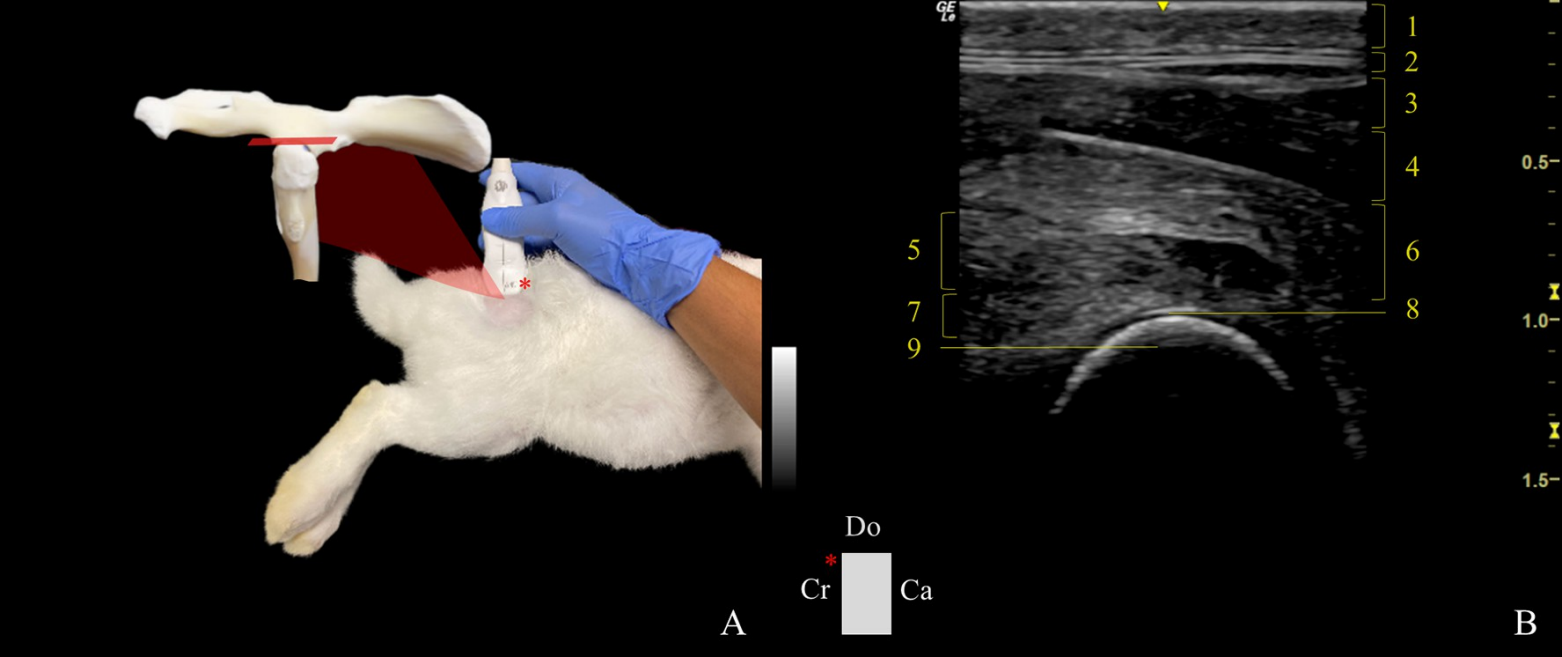
Transducer positioning and resulting ultrasound image obtained in a dorsal approach in the dorsal plane of the right hip joint. A: Rabbit placement in lateral recumbency with the probe at the level of the joint, and corresponding anatomic specimen. The red rectangle and asterisk represent the probe orientation and indicator, respectively. B: Sonoanatomy of the hip: (1) skin and subcutaneous tissue, (2) fascia glutea, (3) gluteofemoralis muscle, (4) gluteus superficialis muscle, (5) gluteus accessorius muscle, (6) gluteus medius muscle, (7) gluteus profundus muscle, (8) articular cartilage of the femoral head, and (9) femoral head. Do: Dorsal, Cr: cranial, and Ca: caudal.

In the **dorsal approach**, the animals were positioned in lateral decubitus with the hindlimb in a neutral position. Hip abduction was necessary to increase the visualization window of the portion of the femoral head covered by the acetabulum, promoting the distraction of the femoral head. In this approach, the planes were described as presented:

*Dorsal plane*. When accessing the dorsal approach dorsal plane (DDP) (Fig 3), the dorsal edge of the greater trochanter (GT) and the point of the sacrum immediately adjacent to the dorsal edge of the GT were anatomical landmarks considered. The probe was positioned parallel to the sacrum and between the sacrum and the GT. The transducer was laterally tilted ~10˚ to visualize the dorsal acetabular rim, and dorsal aspect of the femoral head and neck. After the transducer was slid from the joint to the femoral diaphysis, the observation of the dorsal acetabular rim, dorsal aspect of the femoral neck, and great and third trochanters was possible. The indicator on the probe was pointing cranially.

**Fig 4:**
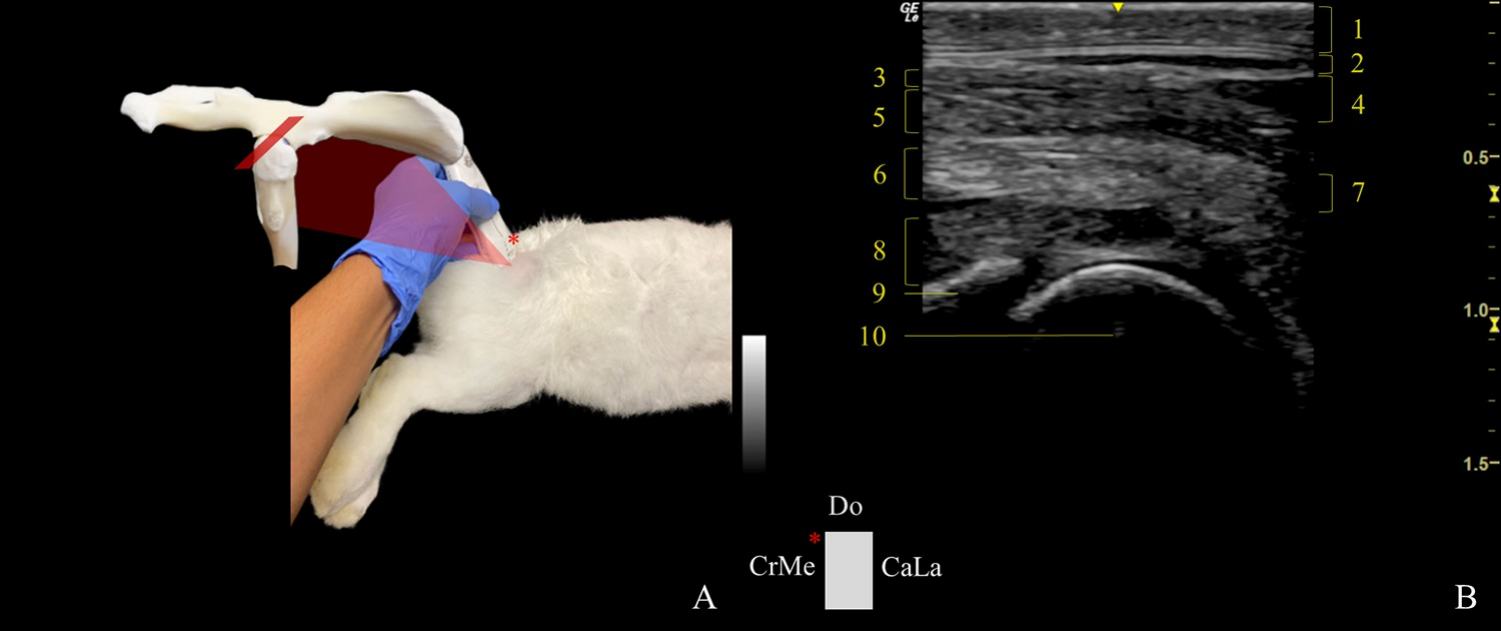
Transducer positioning and resulting ultrasound image obtained in a dorsal approach in the caudolateral-craniomedial oblique plane of the right hip joint. A: Rabbit placement in lateral recumbency with the probe at the level of the joint, and corresponding anatomic specimen. The red rectangle and asterisk represent the probe orientation and indicator, respectively. B: Sonoanatomy of the hip: (1) skin and subcutaneous tissue, (2) fascia glutea, (3) gluteus superficialis muscle, (4) gluteofemoralis muscle, (5) gluteus medius muscle, (6) gluteus accessorius muscle, (7) piriformis muscle, (8) gluteus profundus muscle, (9) acetabulum, and (10) femoral head. Do: dorsal, CrMe: craniomedial, and CaLa: Caudolateral.Ca: caudal, Me: medial., and La: lateral.

*CaudoLateral-CranioMedial oblique plane*. When employing the dorsal approach caudolateral-craniomedial oblique plane (DCaLa-CrMeOP) (Fig 4), the anatomical landmarks deemed necessary were the dorsal edge of the GT, the point of the sacrum immediately adjacent to the dorsal edge of the GT, and the ischial tuberosity, which formed an imaginary triangle. The probe was placed on the side of the triangle formed by the sacrum and the ischial tuberosity, keeping it close to the GT as possible. The transducer was dorsocaudally tilted ~20˚ to assess the caudal edge of the dorsal acetabular rim and dorsal aspect of the femoral head and neck. The indicator was placed obliquely and directed craniomedially.

**Fig 5:**
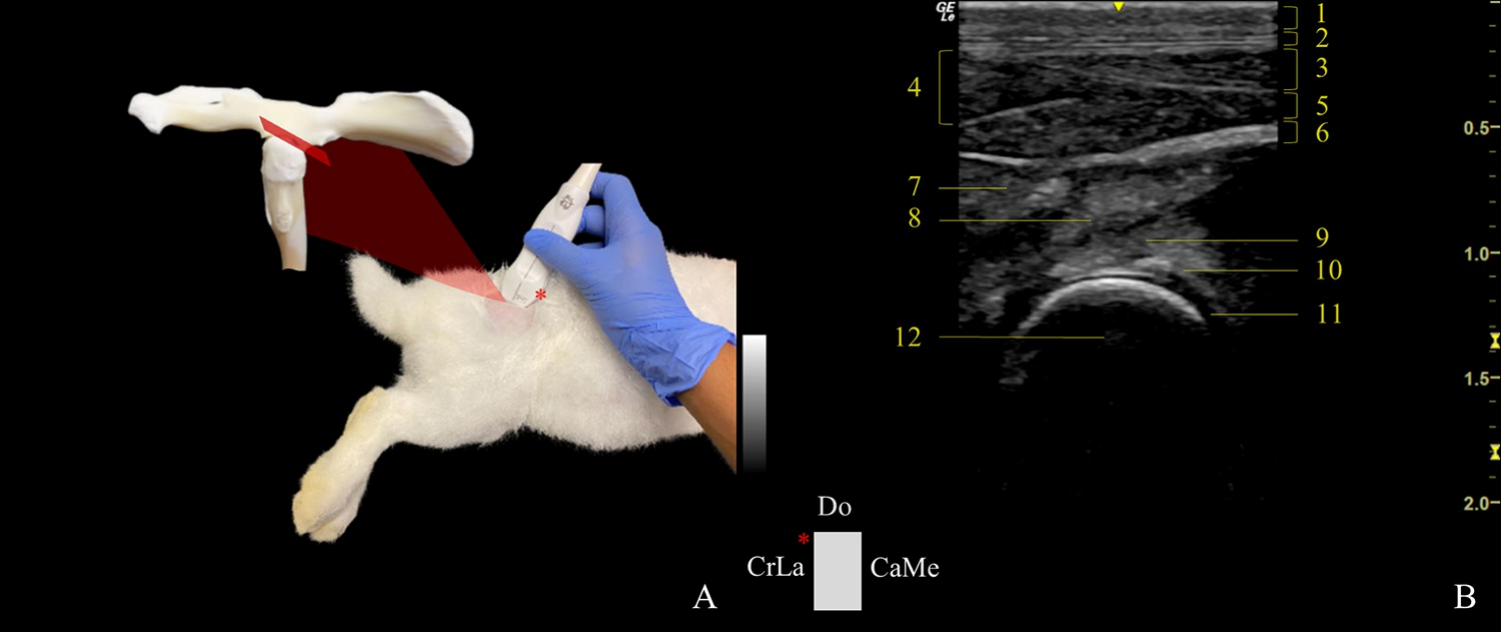
Transducer positioning and resulting ultrasound image obtained in a dorsal approach in the caudomedial-craniolateral oblique plane of the right hip joint. A: Rabbit placement in lateral recumbency with the probe at the level of the joint, and corresponding anatomic specimen. The red rectangle and asterisk represent the probe orientation and indicator, respectively. B: Sonoanatomy of the hip: (1) skin and subcutaneous tissue, (2) fascia glutea, (3) gluteofemoralis muscle, (4) gluteus superficialis muscle, (5) gluteus medius muscle, (6) sciatic nerve, (7) gluteus accessorius muscle, (8) piriformis muscle, (9) gluteus profundus muscle, (10) dorsal acetabular rim, (11) articular cartilage of the femoral head, and (12) femoral head. Do: dorsal, CrLa: craniolateral, and CaMe: caudomedial.

*CaudoMedial-CranioLateral oblique plane*. To perform the dorsal approach caudomedial-craniolateral oblique plane (DCaMe-CrLaOP) (Fig 5), the required anatomical landmarks were the dorsal edge of the GT, the point of the sacrum immediately adjacent to the dorsal edge of the GT, and the iliac wing, which formed an imaginary triangle. The probe was positioned at the side of the triangle formed by the sacrum and iliac wing, keeping it close to the GT as possible. The transducer was ventrocaudally tilted ~20˚ to assess the cranial edge of the dorsal acetabular rim, labrum, and the dorsal aspect of the femoral head and neck. The indicator was oriented obliquely and pointed craniolaterally.

**Fig 6:**
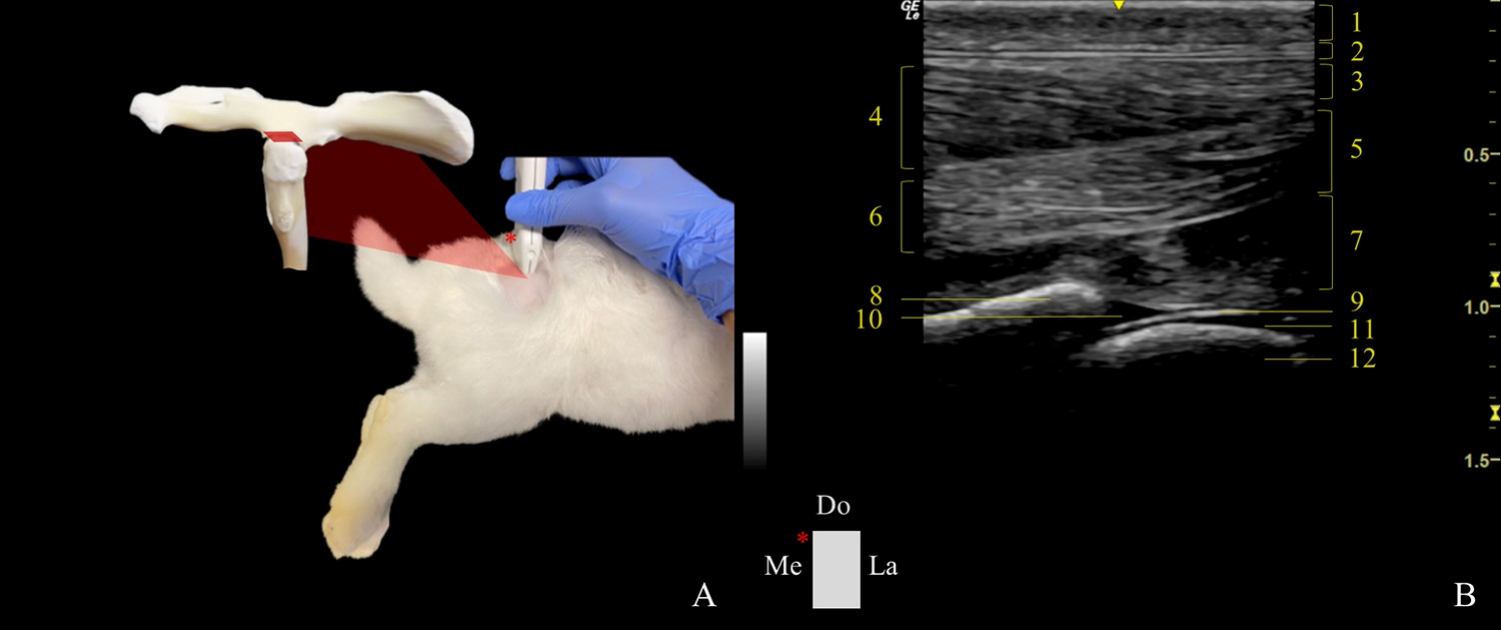
Transducer positioning and resulting ultrasound image obtained in a dorsal approach in the transverse plane of the right hip joint. A: Rabbit placement in lateral recumbency with the probe at the level of the joint, and corresponding anatomic specimen. The red rectangle and asterisk represent the probe orientation and indicator, respectively. B: Sonoanatomy of the hip: (1) skin and subcutaneous tissue, (2) fascia glutea, (3) gluteus superficialis muscle, (4) gluteus medius muscle, (5) gluteus accessorius muscle, (6) piriformis muscle (7) gluteus profundus muscle, (8) acetabulum, (9) dorsal aspect of the articular capsule, (10) dorsal acetabular labrum, (11) articular cartilage of the femoral head, and (12) femoral head. Do: dorsal, Me: medial., and La: lateral.

*Transverse plane*. The dorsal edge of the GT and the point of the sacrum immediately adjacent to the dorsal edge of the GT were anatomical landmarks considered when exploring the dorsal approach transverse plane (DTP) (Fig 6). The probe was positioned longitudinally to the GT, and between the GT and sacrum. The transducer was cranially tilted ~10˚ to visualize the dorsal aspect of the acetabulum, acetabular labrum, femoral head, neck, and joint capsule. The indicator on the probe was facing the acetabulum.

#### Phase II: Application of the US-guided protocol in healthy joints

In the second phase, the US-guided protocol was applied to 11 rabbit cadavers with healthy hips (n = 22 hips) to investigate its feasibility in assessing hip joint morphology. For this purpose, US images of each plane were collected in each hip joint, and image quality for each anatomical structure was assessed and classified as good (3), acceptable (2), poor (1), or non-applicable (0), based on a numerical scoring system [38] (Table 1). In total, fifteen articular or structures were considered in the assessment: the acetabulum (cranial, caudal, ventral, and dorsal aspects, and acetabular labrum), the femoral head (cranial, caudal, ventral and dorsal aspects), femoral neck (ventral and dorsal aspects), joint capsule/ synovium profile, synovial profile, and ligament teres. Each hip joint was examined within 10 minutes and both hips were viewed sequentially. For image analysis, a free DICOM Medical Image Viewer Software (Horos, version 4.0.0 RC5) was used.

**Table 1:** The numerical scoring system used in the ultrasonographic image quality assessment of the hip joint in rabbits, adapted from David et al. [38].

Image Quality	Grading	Anatomical Structures Visualization
Good	3	Articular or periarticular structures clearly identified.
Acceptable	2	Articular or periarticular structures fairly/ not clearly identified.
Poor	1	Articular or periarticular structures poorly identified.
Non-Applicable	0	Articular or periarticular structures not identified.

### *In vivo* study

#### Phase III: Application of the US-guided protocol in osteoarthritic joints

In phase III, the US-guided protocol was applied *in vivo* in osteoarthritic hip joints of 20-week-old male New Zealand white rabbits (5 rabbits, n = 5 hips) which were previously subject to a surgically induced OA and had a confirmed OA diagnosis by CT.

The induction surgery was performed at 6-weeks-old in the left hip joint, and all rabbits were submitted to a similar pre-anaesthetic protocol, using as pre-medication methadone (Semfortam^®^, Eurovet Animal Health BV, Netherlands, at 0.5 mg/Kg, IM) and dexmedetomidine (Sedadex^®^, Le Vet Beheer B.V., Netherlands, at 50 μg/Kg, IM). General anaesthesia was induced with ketamine (Ketamidor^®^, Richter Pharma AG, Austria, at 20 mg/kg, IM) and midazolam (Dormazolan^®^, Le Vet Beheer B.V., Netherlands, at 0.5 mg/kg, IM), and maintained using a surgical mask with 1.5% isoflurane (IsoFlo100%^®^, Zoetis, Portugal) in oxygen. Postoperative pain was managed with meloxicam (Meloxidolor^®^, Le Vet Beheer B.V., Netherlands, at 1.0 mg/kg, SC) for 3 days.

The presence of OA was confirmed by CT in these rabbits at 20-week-old, 14 weeks after the induction surgery. In the pre-anaesthetic protocol, butorphanol (Butomidor^®^, Richter Pharma AG, Austria, at 0.4 mg/Kg, IM) and dexmedetomidine (Sedadex^®^, Le Vet Beheer B.V., Netherlands, at 50 μg/Kg, IM) were used as pre-medication. The induction of general anaesthesia followed the application of ketamine (Ketamidor^®^, Richter Pharma AG, Austria, at 20 mg/kg, IM) in combination with midazolam (Dormazolan^®^, Le Vet Beheer B.V., Netherlands, at 0.5 mg/kg, IM), and maintained with 1.5% isoflurane (IsoFlo100%^®^, Zoetis, Portugal) in oxygen using a facial mask.

After the CT assessment, the protocol US established in phase I was then applied *in vivo* in the left hip joint to characterize the osteoarthritic findings. Images were collected in three planes, the VTP, DDP, and DTP. Subsequently, comparisons were made between images collected from non-traumatized hip joints of rabbit cadavers (phase II) and *in vivo* osteoarthritic rabbit hip joints.

### Statistical analysis

Statistical analysis was performed using commercially available software (SPSS Statistics for Windows, Version 27.0, IBM, USA). The basic features of the data were presented using the median (quartile 25%-75%) and mean (standard deviation (SD)). Data were tested for normality using the Shapiro-Wilk test, and non-parametric variables were analysed by the Kruskal-Wallis test followed by Dunn’s multiple comparisons test. The significance values were adjusted by the Bonferroni correction for multiple tests and the statistical significance was set at *P* < 0.05.

## Results

The mean body weight (BW) of 13 rabbits (n = 26 hips) that were used in phase I and II was 3.63 ± 0.21 kg (mean ± SD), while the mean BW of 5 rabbits (n = 5 hips) used in phase III was 4.33 ± 0.41 kg.

### Validation of the US-guided protocol in cadavers

A total of 22 healthy hips from rabbit cadavers were analysed following the established US-Guided protocol to assess image quality in normal articular or periarticular structures identified in each plane (Table 2). In the VSP, the femoral head acquired a curvilinear hyperechoic silhouette, superimposed by the curvilinear anechogenic cartilage and hyperechogenic joint capsule. In the VTP, the acetabulum, femoral head, and neck were presented as hyperechoic lines with posterior acoustic shadowing. The hyaline cartilage of the femoral head was characterized by a thin homogenous anechoic appearance, interposed between the medium echogenicity line of the capsule and the hyperechoic bone silhouette. The acetabular labrum was depicted as having a homogeneous anechoic triangular shape. Moreover, the sartorius, rectus femoris, iliacus and psoas muscles were defined as having longitudinal/ transverse hypoechoic fibrillar patterns. When accessing the DDP, the dorsal acetabular rim, and dorsal aspect of the femoral head and neck were represented as hyperechoic curvilinear structures. When employing the DCaLa-CrMeOP, the gluteal fascia was depicted as a thin hyperechogenic line and underneath, the gluteus and piriformis muscles appeared hypoechoic with distinct echoic lines which were consistent with strong connective tissue. Furthermore, when performing the DCaMe-CrLaOP, the sciatic nerve is highlighted, appearing with a medium echogenicity. In the DTP, the triangular anechoic shape attained by the acetabular labrum was emphasized.

**Table 2:** Descriptive statistics of the data presented as median (quartile 25%-75%) and mean (standard deviation (SD)) of the image quality assessment in normal articular or periarticular structures visualized in each plane in 11 rabbits (n = 22 hips).

			Ventral Approach				Dorsal Approach							
			Sagittal Plane		Transverse Plane		Dorsal Plane		CaudoLateral-CranioMedial Oblique Plane		CaudoMedial-CranioLateral Oblique Plane		Transverse Plane	

		n (hips)	Median (Quartiles 25%-75%)	Mean (SD)	Median (Quartiles 25%-75%)	Mean (SD)	Median (Quartiles 25%-75%)	Mean (SD)	Median (Quartiles 25%-75%)	Mean (SD)	Median (Quartiles 25%-75%)	Mean (SD)	Median (Quartiles 25%-75%)	Mean (SD)
**Acetabulum**	**Cranial Aspect**	22	2 (2–3) ^a^	2.36 (0.58)	2 (2–3) ^a^	2.32 (0.48)	3 (3–3) ^a^	2.91 (0.29)	0 (0–0) ^b^	0.00 (0.00)	3 (3–3) ^a^	2.82 (0.39)	2 (2–2) ^b^	2.00 (0.00)
	**Caudal Aspect**	22	2 (2–3) ^a^	2.36 (0.58)	2 (2–3) ^a^	2.32 (0.48)	3 (3–3) ^a^	2.86 (0.35)	2.5 (2–3) ^a^	2.50 (0.51)	0 (0–0) ^b^	0.00 (0.00)	2 (2–2) ^b^	2.00 (0.00)
	**Ventral Aspect**	22	2 (2–3) ^a^	2.41 (0.50)	2 (2–3) ^a^	2.45 (0.51)	0 (0–0) ^b^	0.00 (0.00)	0 (0–0) ^b^	0.00 (0.00)	0 (0–0) ^b^	0.00 (0.00)	0 (0–0) ^b^	0.00 (0.00)
	**Dorsal Aspect**	22	0 (0–0) ^b^	0.00 (0.00)	0 (0–0) ^b^	0.00 (0.00)	3 (3–3)	2.91 (0.29)	2.5 (2–3)	2.50 (0.51)	3 (3–3)	2.91 (0.29)	3 (3–3)	2.77 (0.43)
	**Acetabular Labrum**	22	0 (0–0) ^b^	0.00 (0.00)	2 (1–2) ^a^	1.64 (0.66)	0 (0–0) ^b^	0.00 (0.00)	0 (0–0) ^b^	0.00 (0.00)	0 (0–0) ^b^	0.00 (0.00)	2 (1.25–3) ^a^	2.09 (0.81)
**Femoral Head**	**Cranial Aspect**	22	3 (2–3) ^a^	2.68 (0.48)	2 (2–3) ^b^	2.45 (0.51)	3 (3–3) ^a^	2.86 (0.35)	0 (0–0) ^b^	0.00 (0.00)	3 (3–3) ^a^	2.82 (0.39)	3 (3–3) ^a^	2.77 (0.43)
	**Caudal Aspect**	22	3 (2–3) ^a^	2.68 (0.48)	2 (2–3) ^a^	2.45 (0.51)	3 (3–3) ^a^	2.82 (0.39)	3 (2.25–3) ^a^	2.73 (0.46)	0 (0–0) ^b^	0.00 (0.00)	3 (3–3) ^a^	2.77 (0.43)
	**Ventral Aspect**	22	3 (2.5–3) ^a^	2.73 (0.46)	2 (2–3) ^a^	2.59 (0.50)	0 (0–0) ^b^	0.00 (0.00)	0 (0–0) ^b^	0.00 (0.00)	0 (0–0) ^b^	0.00 (0.00)	0 (0–0) ^b^	0.00 (0.00)
	**Dorsal Aspect**	22	0 (0–0) ^b^	0.00 (0.00)	0 (0–0) ^b^	0.00 (0.00)	3 (3–3) ^a^	2.95 (0.21)	3 (2.25–3) ^a^	2.73 (0.46)	3 (3–3) ^a^	2.82 (0.39)	3 (3–3) ^a^	2.95 (0.21)
	**Cartilage**	22	3 (3–3) ^a^	2.68 (0.65)	3 (2–3) ^b^	2.55 (0.51)	3 (3–3) ^a^	2.95 (0.21)	3 (2.25–3) ^a^	2.73 (0.46)	3 (3–3) ^a^	2.77 (0.43)	3 (3–3) ^a^	2.91 (0.29)
**Neck**	**Cranial Aspect**	22	2 (2–2) ^a^	1.95 (0.38)	2 (2–3) ^a^	2.41 (0.50)	0 (0–0) ^b^	0.00 (0.00)	0 (0–0) ^b^	0.00 (0.00)	0 (0–0) ^b^	0.00 (0.00)	0 (0–0) ^b^	0.00 (0.00)
	**Caudal Aspect**	22	0 (0–0) ^b^	0.00 (0.00)	0 (0–0) ^b^	0.00 (0.00)	2 (2–3) ^a^	2.36 (0.49)	2 (2–2) ^a^	2.14 (0.47)	2 (2–2.75) ^a^	2.27 (0.46)	0 (0–0) ^a^	0.00 (0.00)
**Joint Capsule/Synovium Profile**		22	0 (0–0) ^b^	0.00 (0.00)	2 (2–3) ^a^	2.23 (0.61)	0 (0–0) ^b^	0.00 (0.00)	0 (0–0) ^b^	0.00 (0.00)	0 (0–0) ^b^	0.00 (0.00)	3 (3–3) ^a^	2.91 (0.29)
**Synovial Profile**		22	0 (0–0) ^b^	0.00 (0.00)	2 (2–2) ^a^	2.23 (0.61)	0 (0–0) ^b^	0.00 (0.00)	0 (0–0) ^b^	0.00 (0.00)	0 (0–0) ^b^	0.00 (0.00)	3 (3–3) ^a^	2.91 (0.29)
**Ligament Teres**		22	0 (0–0) ^b^	0.00 (0.00)	3 (2–3) ^a^	2.45 (0.67)	0 (0–0) ^b^	0.00 (0.00)	0 (0–0) ^b^	0.00 (0.00)	0 (0–0) ^b^	0.00 (0.00)	0 (0–0) ^b^	0.00 (0.00)
**Total Mean (SD)**		-	-	1.32 (0.27)	-	1.86 (0.44)	-	1.51 (0.17)	-	1.02 (0.19)	-	1.09 (0.16)	-	1.74 (0.21)

The plane with the highest mean rank in each structure was considered for the multiple comparisons test with all other planes.

^a^ or ^b^ represent mean ranks that were statistically significantly different in Dunn’s Multiple Comparison Test with the Bonferroni correction between planes and in each structure.

The VTP was the plane with the highest total mean score in the image quality assessment of normal articular or periarticular structures (1.86 ± 0.44), followed by the DTP (1.74 ± 0.21), DDP (1.51 ± 0.17), and VSP (1.32 ± 0.27). The DCaMe-CrLaOP and DCaLa-CrMeOP showed the lowest total mean scores with 1.09 ± 0.16 and 1.02 ± 0.19, respectively.

Some aspects of the acetabulum, namely the cranial and caudal aspects, were better assessed (higher mean rank) in the DDP, whereas the ventral aspect was better evaluated in the VTP, the dorsal aspect in the DDP and DCaMe-CrLaOP, and the acetabular labrum in the DTP. Regarding the femoral head, DDP enabled a clear visualization of the cartilage and the cranial, caudal and dorsal aspects, while the VSP and the DTP enabled the visualization of the ventral and dorsal aspects, respectively. With respect to the femoral neck, the VTP presented a higher mean rank in the cranial aspect and the DDP in the caudal aspect. The joint capsule/ synovium profile and synovial profile had an improved visualization in the DTP. In the ligament teres assessment, VTP was the only plane capable of a clear detection of this structure. Concerning the number of structures identified in each approach when compared with the planes with the lowest mean rank: in the ventral approach, VTP registered statistical significance in a higher number of structures (10 structures out of 15, 10/15) compared to the VSP (8/15), and, in the dorsal approach, DDP and DTP (8/15) presented a greater number of structures assessed relatively to DCaLa-CrMeOP and DCaMe-CrLaOP (6/15).

Table 3 summarizes Dunn’s Multiple Comparison test considering all data of each plane as a collective unit. To identify the plane with the overall clearest image quality, statistical significance was considered between planes of the same approach. In the ventral approach, VSP and VTP revealed statistically significant differences. In the dorsal approach, DDP and DTP presented statistical differences between DCaLa-CrMeOP and DCaMe-CrLaOP.

**Table 3:** Summary of the Dunn’s Multiple Comparison test in the image quality assessment for each plane in 11 rabbit cadavers (n = 22 hips), not considering each articular or periarticular structure individually but as a collective unit.

Plane1—Plane2	Test Statistic	Standard Error	Standard Test Statistic	Significance*
**Ventral_Sagittal—Ventral_ Transverse**	194,676	41,445	4,697	0,000
**Dorsal_Dorsal—Dorsal_CaudoLateralCranioMedialOblique**	-207,939	41,445	-5,017	0,000
**Dorsal_Dorsal—Dorsal_CaudoMedialCranioLateralOblique**	-169,973	41,445	-4,101	0,001
**Dorsal_Dorsal—Dorsal_Transverse**	-76,733	41,445	-1,851	0,962
**Dorsal_CaudoLateralCranioMedialOblique-Dorsal_CaudoMedialCranioLateralOblique**	-37,967	41,445	-0,916	1,000
**Dorsal_CaudoLateralCranioMedialOblique-Dorsal_Transverse**	-284,673	41,445	-6,869	0,000
**Dorsal_CaudoMedialCranioLateralOblique- Dorsal_Transverse_**	-246,706	41,445	-5,953	0,000

*Significance values have been adjusted by the Bonferroni correction.

### Application of the US-guided protocol *in vivo*

**Fig 7:**
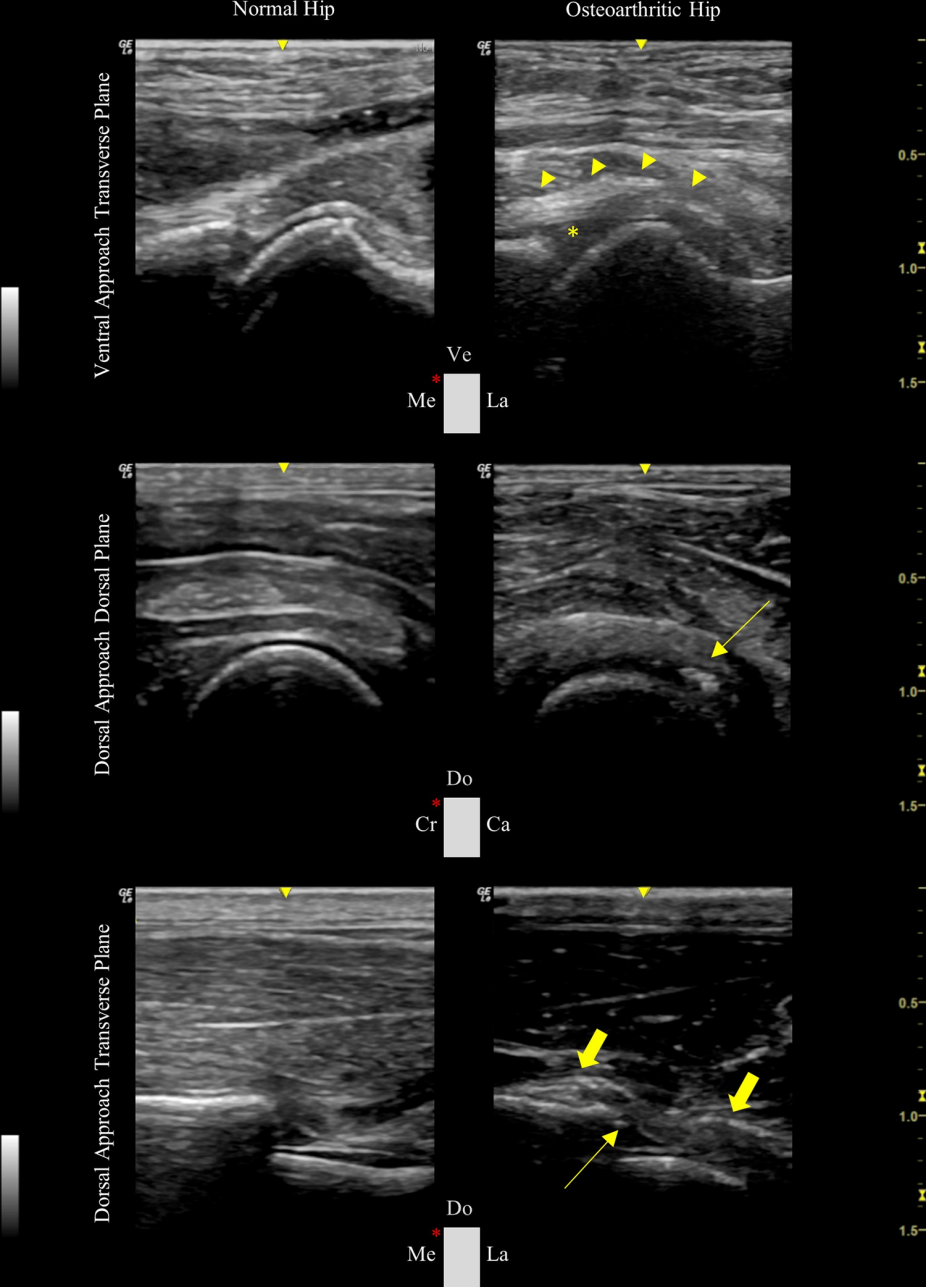
Representative ultrasonographic images of normal and osteoarthritic joints in ventral approach transverse plane and dorsal approach dorsal and transverse planes. The asterisk represents an increased articular volume; the arrowheads, a thickening of the joint capsule/ synovium; the thin arrows, the presence of a hyperechoic bony prominence compatible with an osteophyte, and the large arrows, a heterogeneous and hypertrophic joint capsule/ synovium. Ve: Ventral, Me: medial., La: lateral, Do: dorsal, Cr: cranial, and Ca: caudal.

In this section were compared images from healthy hip joints of rabbit cadavers and OA joints obtained in the three planes which presented the highest total mean score in the image quality assessment in phase II (VTP, DDP, and DTP) (Fig 7). The US imaging revealed distinctive features between articular and periarticular structures of healthy and osteoarthritic hip joints. In osteoarthritic joints, loss of sharpness and normal anechoic echostructure was observed, in addition to irregularities of the cartilage on the superficial and deep margins. The joint capsule/ synovium was recognized with a heterogeneous and hypertrophic silhouette and a higher articular volume compatible with joint effusion was also described. At the femoral or acetabular levels, cortical protrusions consistent with the presence of osteophytes were documented. Overall, injured cartilage was essentially observed in the VTP and DDP, the increased articular volume in the VTP, and a thickened joint capsule/ synovium and osteophytes were only observed in the DDP and DTP.

## Discussion

Due to the relevance of the rabbit as an animal model of human OA [39] and the emerging demand for generating feasible therapeutic approaches to treat hip OA [32], the development of a US-guided protocol to monitor this arthropathy is of utmost importance. Therefore, the present research work aimed to highlight the hip joint’s sonoanatomy, establish a US-guided protocol for the monitorization of the hip joint, and identify OA of the hip in rabbits using the US.

Extensive literature survey does not provide a clear standardized approach to the assessment of the hip by means of ultrasonographic imaging in the field of veterinary sciences, even though some attempts have been described regarding guided injections [30–37]. Consequently, the evidence collected in the literature for other species was transposed and adapted to the rabbit in the first phase of this work. A total of two planes in the ventral approach and four planes in the dorsal approach were described and normal anatomical features of the hip region were reported. Using the US and palpable anatomical landmarks, the coxofemoral joint was easily located in all planes. Overall, the femoral diaphysis, the dorsal edge of the GT, the point of the sacrum immediately adjacent to the dorsal edge of the GT, and the iliac wing were useful landmarks for the probe positioning. In a US examination of the hip joint in dogs, Bergamino et al. [32] provided evidence for the usefulness of the GT and iliac wing as anatomical landmarks. Moreover, the rabbit exhibited some important peculiarities in the hip region when anatomically compared to carnivores, namely the presence of a gluteus accessorius muscle [40] and a third trochanter [41]. Therefore, the pilot stage and gross anatomical dissection in phase I were considered essential for the US-anatomical correlation.

Hip OA is the result of an abnormal stress distribution derived from a poor relationship between the acetabulum and the femoral head [42, 43]. This uneven load distribution is translated into higher contact stress in certain regions. In humans, the regions of greatest clinical significance are the cranial and dorsal aspects of the acetabulum and femoral head [18, 44]. In dogs, cartilage lesions most frequently observed are located around the ligament teres insertion, cranial or caudal aspects of the acetabulum and femoral head, and acetabular labrum [45]. Taking into consideration the preferential locations of the cartilage defects, this work was designed to describe the planes that better define the morphology of the hip joint. VTP was deemed more informative regarding the hip joint sonoanatomy due to the highest total mean score, enabling the identification of a clearer number of structures when compared to the other planes. Also, it was the only plane able to assess the ligament teres integrity. DDP showed the fairest amount of consistency between joint examinations and structures in the image quality assessment, presenting a considerable number of median values of 3. This plane also granted the clearest identification of the cranial and caudal aspects of the acetabulum and femoral head, and the caudal aspect of the femoral neck, which are areas with higher susceptibility of being subject to concentric forces when hip instability is present [45, 46]. Additionally, DCaLa-CrMeOP or DCaMe-CrLaOP offered a further evaluation of the cranial or caudal parts of the acetabulum and femoral head [34] and may be used to confirm preliminary suspicions regarding cartilage lesions in this area in a slightly different US plane. Another area that should be meticulously evaluated is the dorsal aspect of the acetabulum and femoral head [45], which is readily imaged in all planes using the dorsal approach, highlining the DDP and DCaMe-CrLaOP with the highest total mean score for the dorsal aspect of the acetabulum and DDP and DTP for the dorsal aspect of the femoral head. All extent of the ventral acetabular margin or dorsal acetabular rim can be evaluated clearly in the VSP and VTP or DDP and DCaMe-CrLaOP, respectively. Our results suggest that the use of a single plane seems to be insufficient for a complete and detailed evaluation of all articular and periarticular structures. And since no plane is effective in assessing simultaneously the 15 selected structures, it is mandatory to apply other planes complementarily.

In general, the total mean score and the significance found when the structures were analysed individually and collectively assisted in the categorization of the planes according to their clinical relevance. In the ventral approach, VTP, and in the dorsal approach, DDP and DTP were considered superior, being accountable for gathering more relevant data, when compared to VSP and to DCaLa-CrMeOP and DCaMe-CrLaOP, respectively. Moreover, VSP, DCaLa-CrMeOP and DCaMe-CrLaOP did not allow an exclusive and specific evaluation of a given structure, i.e., there were always other planes that due to their higher total mean score admitted a clearer identification of the same structure. As a result, VTP, DDP, and DTP were identified as primary due to their greater clinical relevance, while VSP, DCaLa-CrMeOP and DCaMe-CrLaOP were defined as secondary. Furthermore, the secondary planes may be used as a complement to the primary planes and may be employed according to the daily clinical requirements in examining a certain feature of the hip in additional planes.

According to the Outcome Measures in Rheumatology group (OMERACT), when performing a US scan, a series of osteochondral or synovial tissue findings can be indicative of OA pathology, particularly cartilage lesions, osteophytes, erosion, effusion, and synovial hypertrophy [47]. For instance, articular cartilage lesions can be characterized by deep/ superficial margin irregularities that create an asymmetry in thickness, which are translated in the absence of a homogeneous anechoic appearance [47, 48]. Furthermore, osteophytes or erosion are depicted as bone prominences or disruptions that alter the hyperechoic bone silhouette, respectively [47, 48]. These articular or periarticular changes interfere with the joint integrity and, therefore, a complete US scan of the coxofemoral articulation should comprise the evaluation of the cartilage appearance, the thickness of the joint capsule/ synovium [35], the bone contours, and the presence of effusion/ synovitis [35, 36] in several planes to confirm such changes [47]. Our study corroborated that, despite an increased articular volume compatible with joint effusion/ synovitis being detectable ultrasonographically [35, 49], the synovial fluid and synovium were not normally distinguishable in healthy joints. Additionally, we were able to demonstrate that the primary planes, VTP, DDP, and DTP are capable of spotting cartilage deformities and OA even at an early stage. In our study, osteophytes and capsular hypertrophy were among the lesions most frequently observed. These lesions according to Boulocher et al. [50] are signs of severe OA. Moreover, these findings support the use of US in clinical practice [22, 36] as it allows a definitive diagnosis of this hip arthropathy and a clear definition of its extent. Nevertheless, our results should be confirmed in a sizeable sample and the translation potential to other species, such as humans and dogs which are species described with a substantial prevalence of OA, should be studied [51, 52].

Our study showed that the US-guided protocol produced optimal results, regardless of the limited acoustic window offered by this imaging modality. Its application provided a US characterization of the hip joint in rabbits and supported the clinical use of US in this region. Nevertheless, additional studies in healthy rabbits are required to better define the physiological parameters of the articular or periarticular structures of the hip joint. Moreover, the confirmed applicability of the protocol developed in osteoarthritic joints may offer added insight into the monitoring of OA and instigate future therapeutic research, including in the area of US-guided intra-articular administrations. Although Wang et al. [37] already described the feasibility of US-guided intra-articular injections in the hip joint, further investigation in rabbits is warranted for routine therapeutic purposes. Furthermore, an additional discussion concerning the definition of targeted hip areas in the onset and progression of OA in rabbits is deemed necessary.

The present work was focused on the assessment of the hip joint structural integrity and, therefore, a more detailed research should be performed to address the inflammatory involvement of the OA in the region, namely through the use of Power Doppler. A further limitation is the size of the sample which may have restricted the statistical power of the study. Hence, additional investigation is required to extrapolate the presented US-guided technique to the clinical scenario in a sizeable sample and to study inter- and intra-observer variability.

## Conclusions

In conclusion, the present work illustrated the sonoanatomy of the coxofemoral joint and demonstrated the reliability of the ultrasonographic image acquisition using the US-assisted protocol developed. This novel study provides a sonoanatomical reference for forthcoming therapeutic research and monitoring of OA development, granting the accurate identification of osteophytes or other osseous and cartilaginous defects.

## Supporting information

S1 Data Raw data of the image quality assessment in normal articular or periarticular structures visualized in each plane in 11 rabbits (n = 22 hips). (XLSX)
